# Association of germline variants in telomere maintenance genes (*POT1, TERF2IP, ACD,* and *TERT*) with spitzoid morphology in familial melanoma: A multi-center case series

**DOI:** 10.1016/j.jdin.2023.01.013

**Published:** 2023-01-30

**Authors:** Alisa M. Goldstein, Richard Qin, Emily Y. Chu, David E. Elder, Daniela Massi, David J. Adams, Paul W. Harms, Carla Daniela Robles-Espinoza, Julia A. Newton-Bishop, D. Timothy Bishop, Mark Harland, Elizabeth A. Holland, Anne E. Cust, Helen Schmid, Graham J. Mann, Susana Puig, Miriam Potrony, Llucia Alos, Eduardo Nagore, David Millán-Esteban, Nicholas K. Hayward, Natasa Broit, Jane M. Palmer, Vaishnavi Nathan, Elizabeth G. Berry, Esteban Astiazaran-Symonds, Xiaohong R. Yang, Margaret A. Tucker, Maria Teresa Landi, Ruth M. Pfeiffer, Michael R. Sargen

**Affiliations:** a Division of Cancer Epidemiology and Genetics, National Cancer Institute, Rockville, Maryland; b Department of Dermatology, Hospital of the University of Pennsylvania, Philadelphia, Pennsylvania; c Department of Pathology and Laboratory Medicine, Hospital of the University of Pennsylvania, Philadelphia, Pennsylvania; d Section of Pathological Anatomy, Department of Health Sciences, University of Florence, Florence, Italy; e Experimental Cancer Genetics, The Wellcome Trust Sanger Institute, Hinxton, England; f Department of Pathology, University of Michigan, Ann Arbor, Michigan; g Laboratorio Internacional de Investigación sobre el Genoma Humano, Universidad Nacional Autónoma de México, Campus Juriquilla, Santiago de Querétaro, Qro, Mexico; h Division of Haematology and Immunology, Institute of Medical Research at St James’s, University of Leeds, Leeds, England; i The Daffodil Centre, The University of Sydney, a joint venture with Cancer Council, NSW, Sydney, Australia; j Centre for Cancer Research, Westmead Institute for Medical Research, The University of Sydney, Sydney, Australia; k Sydney School of Public Health, The University of Sydney, Sydney, NSW, Australia; l Melanoma Institute Australia, The University of Sydney, Sydney, NSW, Australia; m John Curtin School of Medical Research, Australian National University, Canberra, ACT, Australia; n Melanoma Unit, Department of Dermatology, Hospital Clínic de Barcelona, IDIBAPS, Barcelona University, Barcelona, Spain; o Centre of Biomedical Research on Rare Diseases (CIBERER), ISCIII, Barcelona, Spain; p Melanoma Unit, Biochemistry and Molecular Genetics Department, Hospital Clínic de Barcelona, IDIBAPS, Barcelona University, Barcelona, Spain; q Pathology Department, Hospital Clínic de Barcelona, Universitat de Barcelona, Barcelona, Spain; r Department of Dermatology, Fundación Instituto Valenciano de Oncología, València, Spain; s School of Medicine, Universidad Católica de València San Vicente Mártir, València, Spain; t QIMR Berghofer Medical Research Institute, Herston, QLD, Australia; u Department of Dermatology, Oregon Health and Science University, Portland, Oregon; v Laboratory of Pathology, Center for Cancer Research, National Cancer Institute, Bethesda, Maryland

**Keywords:** *ACD*, familial melanoma, melanoma, *POT1*, spitzoid melanoma, spitz melanoma, *TERF2IP*, *TERT*, CI, confidence interval, GPV, germline pathogenic variant, OR, odds ratio, TMG, telomere maintenance gene

## Abstract

**Background:**

Spitzoid morphology in familial melanoma has been associated with germline variants in *POT1*, a telomere maintenance gene (TMG), suggesting a link between telomere biology and spitzoid differentiation.

**Objective:**

To assess if familial melanoma cases associated with germline variants in TMG (*POT1*, *ACD*, *TERF2IP*, and *TERT*) commonly exhibit spitzoid morphology.

**Methods:**

In this case series, melanomas were classified as having spitzoid morphology if at least 3 of 4 dermatopathologists reported this finding in ≥25% of tumor cells. Logistic regression was used to calculate odds ratios (OR) of spitzoid morphology compared to familial melanomas from unmatched noncarriers that were previously reviewed by a National Cancer Institute dermatopathologist.

**Results:**

Spitzoid morphology was observed in 77% (23 of 30), 75% (3 of 4), 50% (2 of 4), and 50% (1 of 2) of melanomas from individuals with germline variants in *POT1*, *TERF2IP*, *ACD*, and *TERT*, respectively. Compared to noncarriers (*n* = 139 melanomas), *POT1* carriers (OR = 225.1, 95% confidence interval: 51.7-980.5; *P* < .001) and individuals with *TERF2IP, ACD,* and *TERT* variants (OR = 82.4, 95% confidence interval: 21.3-494.6; *P* < .001) had increased odds of spitzoid morphology.

**Limitations:**

Findings may not be generalizable to nonfamilial melanoma cases.

**Conclusion:**

Spitzoid morphology in familial melanoma could suggest germline alteration of TMG.

**Capsule Summary**

• Germline pathogenic variants in telomere maintenance genes *POT1*, *TERF2IP*, *ACD*, and *TERT* predispose to melanoma.
• Spitzoid morphology is common in familial melanoma cases from individuals with *POT1*, *TERF2IP, ACD,* and *TERT* germline variants. Dermatologists and dermatopathologists should be aware of these associations when ordering familial melanoma gene testing panels.

## Introduction

Approximately 10% of cutaneous melanoma patients have a family history of the disease (familial melanoma).^1^ Germline pathogenic variants (GPVs) in melanoma susceptibility genes *CDKN2A*, *CDK4*, and *BAP1* are identified in up to 40%, 0.7%, and 1.0% of familial melanoma cases, respectively.^1^^,^^2^ More recently, GPV in telomere maintenance genes (TMG) *POT1*, *TERF2IP*, *ACD*, and *TERT* have been observed in up to 6% of melanoma-prone families.^3^, ^4^, ^5^, ^6^, ^7^ *POT1* GPV also predispose to lymphoid and myeloid malignancies, angiosarcoma, and glioma.^8^^,^^9^

*BAP1* GPV carriers commonly develop spitzoid melanocytic neoplasms, characterized by large epithelioid melanocytes with abundant cytoplasm. The biological mechanisms underlying this genotype-histotype association are unknown.^10^^,^^11^ Spitzoid subtype melanomas with complete spitzoid morphology (100% of tumor) and cutaneous melanomas with focal spitzoid morphology (≥25% of tumor) have also been observed in *POT1* GPV carriers, suggesting dysfunctional telomere maintenance as a possible mechanism for spitzoid histology.^12^ Here, we further investigate this hypothesis by evaluating the pathology of familial melanoma cases from individuals with germline variants in TMG.

## Methods

Whole slide images of cutaneous melanomas from individuals with any germline variant in a TMG (*POT1*, *TERF2IP*, *ACD*, and *TERT*) and at least one first, second, or third degree relative with melanoma, were requested during the virtual Melanoma Genetics Consortium (GenoMEL) annual meeting on July 27, 2020. Images were provided to the National Cancer Institute by research groups conducting familial melanoma studies in the United States (1 group), Europe (3 groups), and Australia (2 groups). Collaborators submitted all melanoma cases they had access to from variant carriers, which were independently reviewed by 4 dermatopathologists (MRS, DEE, EYC, and DM) with expertise in melanocytic lesions, who were blinded to clinical information and the specific gene altered. Each dermatopathologist reported histologic subtype and the percentage of tumor cells exhibiting spitzoid morphology, defined as large epithelioid or spindled melanocytes with a cytoplasm to nuclear ratio of greater than or equal to 1. To be consistent with our previous study on this topic, melanomas were classified as spitzoid if at least 3 of 4 dermatopathologists reported spitzoid morphology in 25% or more of the tumor cells.^12^

Logistic regression was used to estimate odds ratios (ORs) and 95% confidence intervals (CIs) for spitzoid morphology and subtype in variant carriers compared to unmatched noncarriers (United States, Europe). All noncarriers had whole exome sequencing performed to exclude GPV in melanoma predisposition genes (*CDKN2A, CDK4, BAP1, POT1, TERT, ACD,* and *TERF2IP*). Noncarrier cases (all cutaneous melanomas) were previously reviewed by a single dermatopathologist (MRS),^12^ and were from individuals with at least one first, second, or third degree relative with melanoma (same as variant carriers). CIs accommodated within person and within family correlations by using a generalized estimating equation approach with a working independence covariance matrix. Analyses were performed in R Studio and SAS 9.4 (SAS Institute Inc).

Familial melanoma studies submitting whole slide images to the National Cancer Institute had local institutional review board or ethics review committee approval.

## Results

Four dermatopathologists reviewed the histology of melanomas from individuals with germline variants in *POT1* (*n* = 30 melanomas, 17 individuals), *TERF2IP* (*n* = 4 melanomas, 3 individuals), *ACD* (*n* = 4 melanomas, 2 individuals), and *TERT* (*n* = 2 melanomas, 2 individuals). Clinical information on variant carriers (28 women [70%], 24 from Europe [60%]) and noncarriers (70 women [50.4%], 81 from Europe [58%]) is available in Table I. Similar proportions of melanomas in the 2 groups were invasive (variant carriers vs noncarriers: 87.5% vs 79.1%, two-sided Fisher’s exact *P* = 0.36), ulcerated (variant carriers vs non-carriers: 15.0% vs 19.1%, two-sided Fisher’s exact *P* = 0.38), and had a mitotic rate of ≥1 mitosis per square millimeter (variant carriers vs non-carriers: 42.9% vs 33.6% for invasive melanomas, two-sided Fisher’s exact *P* = 0.42).

**Table I:** Clinical and histologic characteristics of familial melanoma cases

Characteristic	Noncarrier∗^,^¶	Germline variants in telomere maintenance genes			
		*ACD*¶	*TERF2IP*¶	*TERT*¶	*POT1*¶
No. of families	68	2	3	2	16
No. of individuals	132	2	3	2	17
No. of cases (%)	139 (100)	4 (100)	4 (100)	2 (100)	30 (100)
Country, No. (%)					
United States	58 (41.7)	0	0	0	1 (3.3)
Europe†	81 (58.3)	0	2 (50)	1 (50)	21 (70.0)
Australia	0	4 (100)	2 (50)	1 (50)	8 (26.7)
Female sex, No. (%)	70 (50.4)	4 (100)	1 (25)	1 (50)	22 (73.3)
Median (range) age at diagnosis, y	49 (15-91)	55 (50-56)	41 (20-59)	29.5 (28-31)	52 (35-77)
Body site, No. (%)					
Head and neck	18 (12.9)	0	0	0	1 (3.3)
Trunk	56 (40.3)	2 (50)	1 (25)	2 (100)	13 (43.3)
Upper extremities	26 (18.7)	1 (25)	3 (75)	0	5 (16.7)
Lower extremities	31 (22.3)	1 (25)	0	0	11 (36.7)
Unknown	8 (5.8)	0	0	0	0
Subtype, No. (%)‡					
Superficial spreading melanoma	104 (74.8)	4 (100)	3 (75)	1 (50)	21 (70)
Nodular melanoma	12 (8.6)	0	1 (25)	0	4 (13.3)
Spitzoid melanoma	2 (1.4)	0	0	1 (50)	4 (13.3)
Lentigo maligna melanoma	17 (12.2)	0	0	0	0
Acral melanoma	4 (2.9)	0	0	0	1 (3.3)
Spitzoid morphology, No. (%)§	2 (1.4)	2 (50)	3 (75)	1 (50)	23 (76.7)
Breslow thickness (mm), No. (%)‖					
in situ	29 (20.9)	0	0	0	5 (16.7)
0.1-0.7	59 (42.4)	3 (75)	2 (50)	2 (100)	10 (33.3)
0.8-2.0	29 (20.9)	1 (25)	2 (50)	0	6 (20)
2.1-4.0	14 (10.1)	0	0	0	8 (26.7)
>4.0	8 (5.8)	0	0	0	1 (3.3)
Ulceration‖	13 (19.1)	0	1 (25)	0	5 (16.7)
No. (%) of invasive melanomas‖	110 (100)	4 (100)	4 (100)	2 (100)	25 (100)
Mitotic rate (#/mm^2^) for invasive melanomas					
0	73 (66.4)	4 (100)	3 (75)	2 (100)	11 (44)
1-5	27 (24.5)	0	1 (25)	0	11 (44)
>5	10 (9.1)	0	0	0	3 (12)

*NCI*, National Cancer Institute.

∗ Pathogenic variants in melanoma susceptibility genes (*CDKN2A*, *CDK4*, *BAP1*, *POT1*, *TERT*, *ACD*, *TERF2IP*) were excluded by whole exome sequencing analysis.

† All European noncarrier cases were from Italy (*n* = 81). European variant carrier cases were from Spain (*n* = 2 from *TERF2IP* variant carriers; *n* = 1 from a *TERT* variant carrier; *n* = 12 from *POT1* variant carriers) and the United Kingdom (*n* = 9 from *POT1* variant carriers).

‡ All melanomas from individuals with germline variants in telomere maintenance genes (*POT1*, *TERF2IP*, *ACD*, and *TERT*) were independently reviewed by 4 dermatopathologists (MRS, EC, DM, and DEE), and melanoma subtype was based on the majority opinion of the reviewers. Histologic subtype for noncarrier cases was assessed by a single dermatopathologist (MRS).

§ Spitzoid morphology was defined as large epithelioid or spindled melanocytes with a cytoplasm to nuclear ratio of greater than or equal to 1. Melanomas from individuals with germline variants in telomere maintenance genes (*POT1*, *TERF2IP*, *ACD*, and *TERT*) were classified as spitzoid if at least 3 of the 4 dermatopathologists (MRS, EC, DM, and DEE) reported spitzoid morphology in 25% or more of the tumor cells. Dermatopathologist agreement on spitzoid classification was slight to moderate (vs NCI dermatopathologist [MRS]: non-NCI reviewer 1, kappa = 0.42; non-NCI reviewer 2, kappa = 0.16; and non-NCI reviewer 3, kappa = 0.38), consistent with a previous study (PubMed ID: 34757982) evaluating interobserver agreement for spitzoid classification. Spitzoid morphology in noncarrier cases was assessed by a single dermatopathologist (MRS).

‖ Information was ascertained from pathology reports and confirmed by histopathology review (MRS).

¶ All noncarriers were white. All variant carriers were white except for one individual with a germline *TERF2IP* variant with unknown race.

Spitzoid morphology involving at least 25% of the tumor was observed by at least 3 dermatopathologists in 77% (23 of 30), 75% (3 of 4), 50% (2 of 4), and 50% (1 of 2) of melanomas from individuals with germline variants in *POT1*, *TERF2IP*, *ACD*, and *TERT*, respectively. Furthermore, 13% (4 of 30) of melanomas from *POT1* carriers and 50% (1 of 2) from *TERT* carriers were completely spitzoid, and therefore, were classified as spitzoid subtype melanomas. The median age at diagnosis for a melanoma with spitzoid morphology was 51.5 years (range: 20-77 years) for the variant carrier group (all genes including *POT1*) and 52 years (range: 35-77 years) for *POT1* variant carriers (Table I).

Compared to noncarriers, individuals with germline *POT1* variants had a 10.5-fold increased odds (95% CI: 2.1-51.7; *P* = .004) of developing a spitzoid subtype melanoma (complete spitzoid morphology) and a 225.1-fold increased odds (95% CI: 51.7-980.5; *P* < .001) of having a melanoma with spitzoid morphology involving at least 25% of the tumor. Restricting the analysis to cases from individuals with likely pathogenic or pathogenic variants in *POT1* based on the American College of Medical Genetics and Genomics and Association for Molecular Pathology guidelines for the interpretation of sequence variants also yielded similar results (OR [spitzoid subtype] = 12.1, 95% CI: 2.2-65.7, *P* = .004; OR [spitzoid morphology] = 205.5, 95% CI: 8.7-1089.9, *P* < .001). *BAP1* germline status was unknown in 10 cases from *POT1* carriers and excluding these cases did not impact associations (Table II).

**Table II:** Odds of spitzoid morphology and subtype among individuals with germline variants in telomere maintenance genes

Group	Spitzoid morphology∗			Spitzoid subtype†		
	No. (%)	OR (95% CI)‡	*P*-value	No. (%)	OR (95% CI)‡	*P*-value
Noncarriers (*N* = 139)§	2 (1.4)	Reference		2 (1.4)	Reference	
Any *POT1* variant (*N* = 30)	23 (76.7)	225.1 (51.7-980.5)	<.001	4 (13.3)	10.5 (2.1-51.7)	.004
Likely pathogenic or pathogenic *POT1* variant (*N* = 20)‖	15 (75.0)	205.5 (8.7-1089.9)	<.001	3 (15.0)	12.1 (2.2-65.7)	.004
Any *POT1* variant from individual with negative testing for germline *BAP1* variants (*N* = 20)	17 (85.0)	388.2 (69.2- 2177.5)	<.001	2 (10)	7.6 (1.0-55.6)	.046
Any *ACD, TERF2IP,* or *TERT* variant (*N* = 10)¶	6 (60.0)	82.4 (21.3-494.6)	<.001	1 (10.0)	8.7 (2.1-51.7)	.004

*ACMG*, American College of Medical Genetics and Genomics; *AMP*, Association for Molecular Pathology.

∗ Spitzoid morphology was defined as large epithelioid or spindled melanocytes with a cytoplasm to nuclear ratio of greater than or equal to 1. Melanomas from individuals with germline variants in telomere maintenance genes (*POT1*, *TERF2IP*, *ACD*, and *TERT*) were classified as spitzoid if at least 3 of the 4 dermatopathologists (MRS, EC, DM, and DEE) reported spitzoid morphology in 25% or more of the tumor cells. Spitzoid morphology for noncarrier cases was assessed by a single dermatopathologist (MRS).

† All melanomas from individuals with germline variants in telomere maintenance genes (*POT1*, *TERF2IP*, *ACD*, and *TERT*) were independently reviewed by 4 dermatopathologists (MRS, EC, DM, and DEE), and melanoma subtype was based on the majority opinion of the reviewers. Histologic subtype for noncarrier cases was assessed by a single dermatopathologist (MRS).

‡ Confidence intervals accommodated within person and within family correlations by using a generalized estimating equation approach with a working independence covariance matrix.

§ The noncarrier group includes the United States and Italian familial melanoma cases from individuals with no identifiable germline pathogenic variants in melanoma predisposition genes (*CDKN2A*, *CDK4*, *BAP1*, *POT1*, *TERT*, *ACD*, and *TERF2IP*) based on whole exome sequencing analysis.

‖ Variant classification based on ACMG and AMP standards and guidelines for the interpretation of sequence variants (PubMed ID: 25741868).

¶ No variants were predicted to be likely pathogenic or pathogenic based on ACMG/AMP standards and guidelines for the interpretation of sequence variants (PubMed ID: 25741868). ACMG/AMP predictions for this group included variants of uncertain significance (*n* = 5 cases) and benign variants (*n* = 5 cases). All variant carriers had at least one first, second, or third degree relative with melanoma.

**Fig 1:**
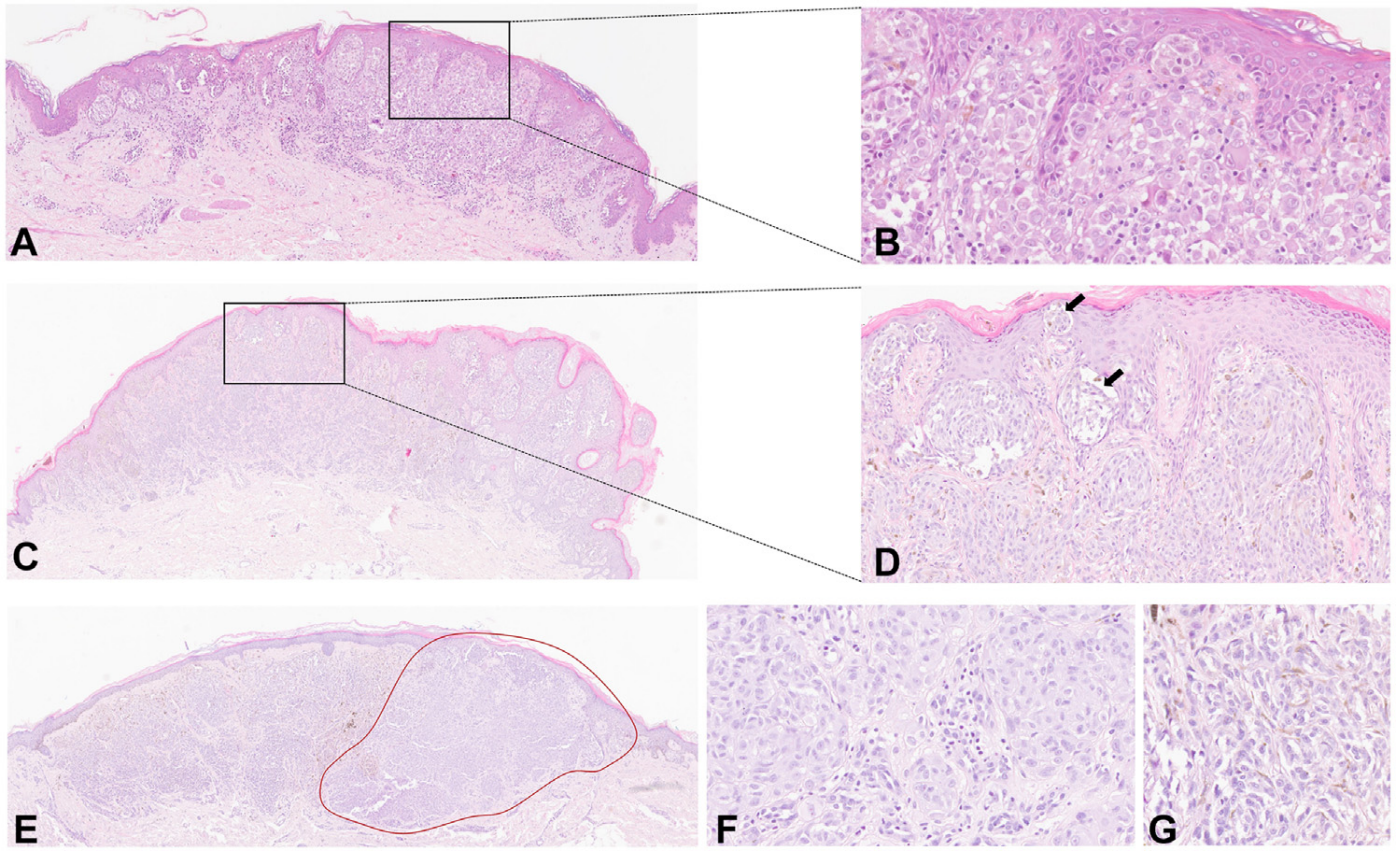
Cutaneous melanomas exhibiting spitzoid morphology from individuals with germline *POT1* variants. **A,** Low magnification (4.9×) of spitzoid subtype melanoma (case 14-B) from an individual with germline *POT1* p.Asp598Serfs∗22 variant. **B,** Higher magnification (25.3×) of case 14-B shows atypical melanocytes with abundant eosinophilic cytoplasm consistent with spitzoid morphology. **C,** Low magnification (2.9×) of spitzoid subtype melanoma (case 3) from an individual with germline *POT1* c.1164-1G>A splice acceptor variant. **D,** Higher magnification (21.1×) of case 3 shows epidermal and dermal nests of epithelioid and spindled melanocytes with abundant cytoplasm; some junctional nests show separation from the surrounding epidermis (*arrows*), which is a feature of Spitz tumors. **E,** Low magnification (3.7×) of a melanoma (case 9) from an individual with germline *POT1* p.Lys427Arg variant; the tumor exhibits areas of spitzoid (*outlined in red*) and nonspitzoid differentiation. **F,** Higher magnification (40×) photomicrograph of spitzoid area in case 9 showing enlarged epithelioid melanocytes with abundant eosinophilic cytoplasm. **G,** Higher magnification (40×) photomicrograph of nonspitzoid area in case 9 showing epithelioid and spindled melanocytes that lack abundant cytoplasm.

**Fig 2:**
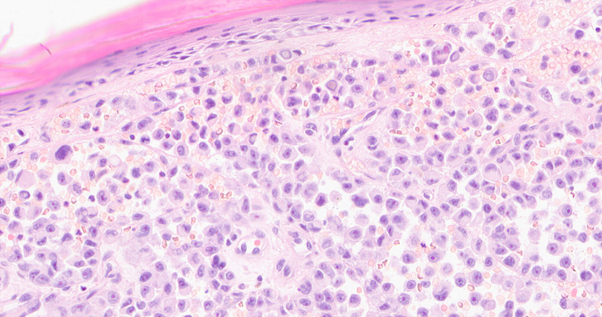
Cutaneous melanoma exhibiting spitzoid morphology with acantholytic features from individual with germline *POT1* variant. Case 6 is from an individual with a germline *POT1* variant (p.Arg117His) and pathology shows a sheet-like proliferation of spitzoid cells with loss of cohesion, consistent with an acantholytic growth pattern.

**Fig 3:**
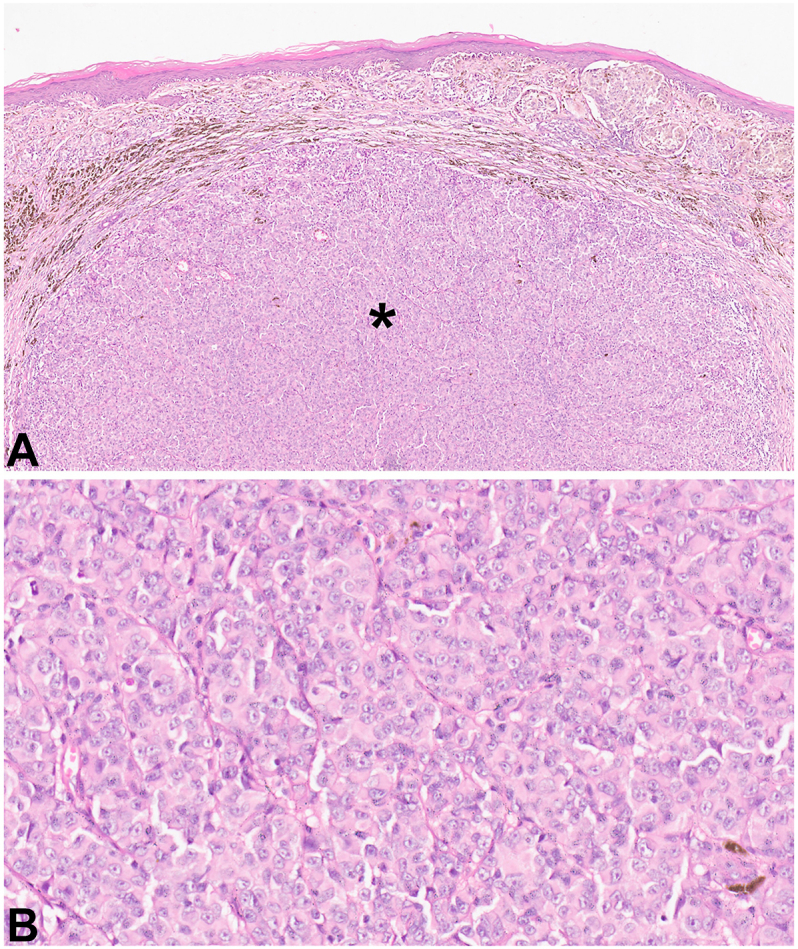
Cutaneous melanoma exhibiting spitzoid morphology in the dermis from individual with germline *POT1* variant. **A,** Case 1 is from an individual with a germline variant in *POT1* (p.Arg137His) and pathology shows a superficial spreading growth pattern (nesting, pagetoid scatter, and increased pigmentation) in the epidermis and an invasive component with spitzoid morphology (*asterisk*). **B,** Higher magnification (40×) of spitzoid area in case 1.

**Fig 4:**
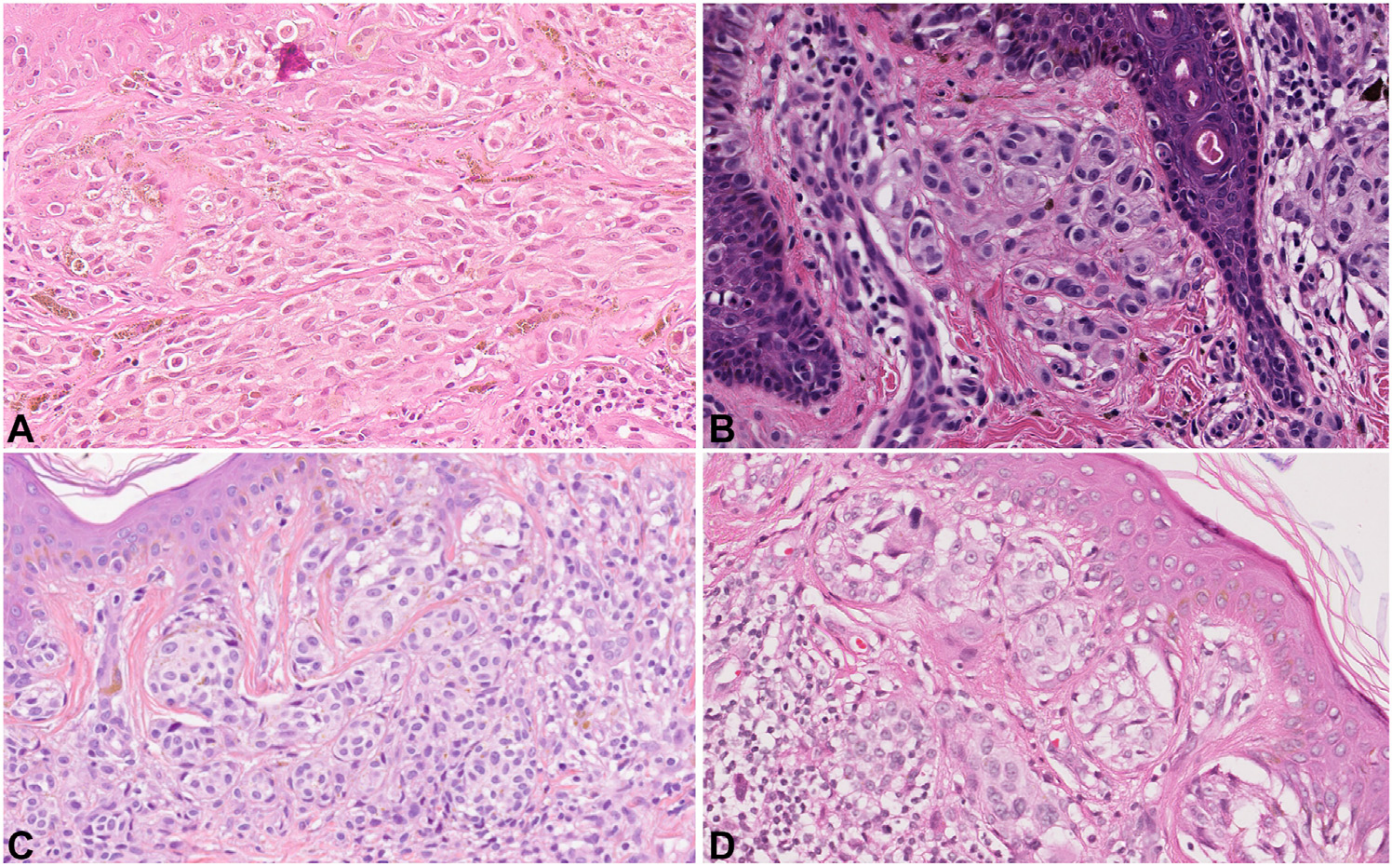
Cutaneous melanomas exhibiting spitzoid morphology from individuals with *TERT*, *TERF2IP*, and *ACD* variants. **A,** Case 24 is from an individual with a germline variant in *TERT* (p.Ala202Thr) and pathology shows nests and sheets of large epithelioid melanocytes with abundant eosinophilic cytoplasm consistent with spitzoid morphology (40× magnification). **B,** Case 18 is from an individual with a germline variant in *TERF2IP* (p.Glu304del) and pathology shows nests and single cells of melanocytes with abundant eosinophilic cytoplasm in the papillary dermis consistent with spitzoid morphology (40× magnification). **C,** Case 20-A is from an individual with a germline variant in *TERF2IP* (p.Pro285Ser) and pathology shows a compound melanocytic lesion with epidermal and dermal nests of epithelioid melanocytes with abundant eosinophilic cytoplasm consistent with spitzoid morphology (40× magnification). **D,** Case 21-C is from an individual with a germline variant in *ACD* (p.Val432Ala) and pathology shows epidermal and dermal nests of melanocytes with abundant eosinophilic cytoplasm, consistent with spitzoid morphology, and a dense lymphocytic infiltrate (40× magnification).

Spitzoid morphology was also more prevalent in melanomas from individuals with *TERF2IP, ACD,* and *TERT* variants compared to noncarriers (OR = 82.4, 95% CI: 21.3-494.6; *P* < .001) (Table II). Representative photomicrographs of spitzoid morphology in cutaneous melanomas from individuals with germline *POT1* variants are shown in Fig 1, Fig 2, Fig 3. Additional photomicrographs of spitzoid morphology in cutaneous melanomas from individuals with germline variants in other TMG are shown in Fig 4.

The American College of Medical Genetics and Genomics and Association for Molecular Pathology predictions for variant pathogenicity are provided in Table III.

**Table III:** Classification of variants using ACMG/AMP criteria

Cases	Gene	Genomic location (human genome reference GRCh38)	Transcripts	Nucleotide change	Amino acid change	ClinVar classification‖	ClinVar ID	dbSNP	ACMG/AMP variant classification‖	ACMG/AMP criteria
1∗	*POT1*	chr7: 124863486	NM_015450.3 ENST00000357628.8	c.410G>A	p.Arg137His	VUS	139523	rs587777475	LP	PM2, PS3, PS4
2∗	*POT1*	chr7: 124851918	NM_015450.3 ENST00000357628.8	c.903G>T	p.Gln301His	LB; B	475112	rs116916706	LB	PM2, BS2, BP6
3∗,5∗	*POT1*	chr7: 124841179	NM_015450.3 ENST00000357628.8	c.1164-1G>A	splice acceptor	LP; P	475026	rs866612394	P	PVS1, PM2, PP5
4∗	*POT1*	chr7: 124842843	NM_015450.3 ENST00000357628.8	c.1127A>G	p.Gln376Arg	Conflicting (VUS; LB)	475020	rs143635917	VUS	PM2, BP6
6∗	*POT1*	chr7: 124863546	NM_015450.3 ENST00000357628.8	c.350G>A	p.Arg117His	VUS	654409	rs1385542313	VUS	PM2, PM5
7∗, 12†, 13‡	*POT1*	chr7: 124858989	NM_015450.3 ENST00000357628.8	c.670G>A	p.Asp224Asn	VUS	139527	rs202187871	LP	PM2, PP3, PS3, PS4
8∗	*POT1*	chr7: 124841131	NM_015450.3 ENST00000357628.8	c.1211G>T	p.Gly404Val	B	475029	rs35536751	B	BA1, BS1, BS2, BP4, BP6
9∗	*POT1*	chr7: 124841062	NM_015450.3 ENST00000357628.8	c.1280A>G	p.Lys427Arg	Not in ClinVar			VUS	PM2, BP4
10‡	*POT1*	chr7: 124870933	NM_015450.3 ENST00000357628.8	c.233T>C	p.Ile78Thr	VUS	475073	rs947005337	LP	PM2, PS4
11-A-F‡	*POT1*	chr7: 124825358	NM_015450.3 ENST00000357628.8	c.1687-1G>A	splice acceptor	Conflicting (VUS; risk factor)	139521	rs587777473	P	PVS1, PM2, PS4
14-A-G‡	*POT1*	chr7: 124825252	NM_015450.3 ENST00000357628.8	c.1792G>A	p.Asp598Serfs∗22	Not in ClinVar		rs1487204320	LP	PM2, PS3
15-A-C§	*POT1*	chr7: 124840979	NM_015450.3 ENST00000357628.8	c.1363A>G	p.Ile455Val	VUS	475039	rs776965979	VUS	PM2
16,17‡	*POT1*	chr7: 124851884	NM_015450.3 ENST00000357628.8	c.937G>A	p.Asp313Asn	VUS	1359398	rs770779418	VUS	PM2, BP4
18‡	*TERF2IP*	chr16: 75656305	NM_018975.4 ENST00000300086.5	c.910_912delGAA	p.Glu304del	Not in ClinVar		rs747026590	VUS	PM2, BP3
19‡	*TERF2IP*	chr16: 75656381	NM_018975.4 ENST00000300086.5	c.970A>G	p.Lys324Glu	Not in ClinVar		rs4888444	B	BS1, BS2, BP4
20-A-B§	*TERF2IP*^e^	chr16: 75656264	NM_018975.4 ENST00000300086.5	c.853C>T	p.Pro285Ser	Not in ClinVar		rs756850727	VUS	PM2, BP4
21-A-C‡, 22‡	*ACD*	chr16: 67657765	NM_001082486.2 ENST00000620761.6	c.1295T>C	p.Val432Ala	B	1167321	rs6979	B	BA1, BS2, BP4, BP6
23‡	*TERT*	chr5: 1254479	NM_198253.3 ENST00000310581.10	c.3184G>A	p.Ala1062Thr	Conflicting (VUS; LB; B)	39121	rs35719940	VUS	PM2, BP3
24‡	*TERT*	chr5: 1294282	NM_198253.3 ENST00000310581.10	c.604G>A	p.Ala202Thr	Conflicting (VUS; LB; B)	12729	rs121918661	VUS	PP2, BS2, BP6

*ACMG*, American College of Medical Genetics and Genomics; *AMP*, Association for Molecular Pathology; *B*, benign; *LB*, likely benign; *LP*, likely pathogenic; *P*, pathogenic; *VUS*, variant of uncertain significance.

∗ Germline variant identified by panel testing of the following genes: *CDKN2A*, *CDK4*, and *POT1*.

† Germline *POT1* variant identified by Sanger sequencing of the gene. Germline whole genome sequencing of a sibling of case 12 with the family *POT1* variant that did not identify variants in other melanoma susceptibility genes including *BAP1*.

‡ Germline variant identified by whole exome/genome sequencing analysis.

§ Germline variant identified by panel testing of the following genes: *CDKN2A, CDK4, BAP1, MITF, POT1, ACD, TERF2IP, TERT* promoter*, BRCA1, BRCA2, MSH2,* and *MSH6*.

‖ ClinVar and ACMG and AMP classifications.

## Discussion

Identification of individuals with GPV in TMG is critically important for counseling and cancer screening. Our analysis identified spitzoid morphology (≥25% of tumor) in 77% of cutaneous melanomas from *POT1* carriers, consistent with a recent case series from our group where 60% of cutaneous melanomas from *POT1* carriers exhibited this histology.^12^ In the current study, all melanomas exhibiting spitzoid morphology from *POT1* carriers occurred in adulthood (range: 35-77 years), although spitzoid subtype melanomas with complete spitzoid morphology (100% of tumor) have been reported in *POT1* carriers as young as age 16 years.^12^ We also identified spitzoid morphology in melanomas from individuals with variants in TMG other than *POT1* (*TERF2IP*, *ACD*, and *TERT*). All cases classified as having spitzoid morphology were independently observed to have this histologic finding by at least 3 of 4 dermatopathologists suggesting that spitzoid cells can be differentiated from other morphologies.

*BAP1* variants have also been associated with spitzoid pathology.^10^ Inactivation of *BAP1* increases *TERT* expression and telomere elongation, suggesting that it may induce spitzoid differentiation via dysfunctional telomere maintenance.^13^ In our study, *BAP1* variants were not detected in any individuals with *TERF2IP*, *ACD*, or *TERT* variants. Furthermore, exclusion of cases from *POT1* carriers with unknown *BAP1* germline status did not significantly impact associations.

Our study findings support the inclusion of TMG (*POT1*, *TERF2IP*, *ACD*, and *TERT*) on genetic testing panels when a familial melanoma case exhibits spitzoid morphology in at least 25% of the tumor cells. Since genetic testing is most commonly ordered by dermatologists, oncologists, or geneticists who evaluate melanoma-prone families, dermatopathologists should report the presence (≥25% of tumor) or absence of spitzoid morphology in the synoptic or microscopic description sections of all melanoma pathology reports regardless of the melanoma subtype (superficial spreading, nodular, lentigo maligna, spitzoid, acral, and desmoplastic) or family medical history, which could be incomplete or unavailable at the time of biopsy. Routine reporting of this information could assist with identifying individuals with GPVs in TMG, and detecting these variants will inform cancer surveillance for the patient, and possibly family members, based on the associated cancer risks.

## Limitations

Although this case series is the largest histopathologic assessment of cutaneous melanomas from individuals with germline variants in TMG, our study ascertained cases from GenoMEL melanoma-prone families, and therefore, the study findings may not be applicable to nonfamilial melanoma cases. Additionally, while cases were independently reviewed by multiple expert dermatopathologists, confirmation bias cannot be excluded. Therefore, case-control studies are needed to confirm the observed findings in this study and assess interobserver agreement for spitzoid classification. Noncarrier cases were reviewed by a single dermatopathologist and were not matched by country, which could have also impacted association estimates. However, previous GenoMEL studies of familial melanoma, involving histopathology review by multiple expert pathologists, did not identify spitzoid morphology in *CDKN2A* carrier and noncarrier familial melanomas.^14^^,^^15^ Lastly, tissue was not available for cases with spitzoid morphology to evaluate for somatic alterations (ie, *HRAS* variants and gene fusions involving *ALK*, *ROS1*, *NTRK1*, *NTRK3*, *MET*, *RET*, *BRAF*, *MAP3K8*, and *CRTC1::TRIM11*) that have been associated with pediatric spitzoid tumors.^16^^,^^17^ Therefore, tumor studies are needed to understand the biological mechanisms linking dysfunctional telomere maintenance to spitzoid differentiation in familial melanoma cases.

## Conclusions

In conclusion, we identified that a high proportion of cutaneous melanomas from individuals with germline variants in TMG exhibit spitzoid morphology. Therefore, panel testing for *POT1*, *TERF2IP*, *ACD*, and *TERT* should be considered when familial melanoma cases exhibit spitzoid differentiation.

## Conflicts of interest

None disclosed.
